# Kein Vorteil für die lokale Kontrolle nach Dosiseskalation der Strahlentherapie bei Patienten mit Ösophaguskarzinom nach einer definitiven Radiochemotherapie: randomisierte Phase-III-Studie ARTDECO

**DOI:** 10.1007/s00066-022-01906-5

**Published:** 2022-02-15

**Authors:** Emmanouil Fokas, Daniel Martin, Claus Rödel

**Affiliations:** 1 Klinik für Strahlentherapie und Onkologie, Universitätsklinikum Frankfurt, Goethe-Universität Frankfurt, Theodor-Stern-Kai 7, 60590 Frankfurt/M., Deutschland; 2 Frankfurt/M., Deutschland

## Hintergrund

In der randomisierten, klinischen Phase-III-Studie ARTDECO wurde bei Patient*innen mit lokal fortgeschrittenem Ösophaguskarzinom die Rolle der Radiotherapiedosiseskalation auf den Primärtumor im Hinblick auf die lokale Tumorkontrolle nach definitiver Radiochemotherapie (RCT) untersucht.

## Methode

Patient*innen mit funktionell inoperablem und/oder irresektablem Ösophaguskarzinom wurden eingeschlossen. Die Randomisierung erfolgte in einen Arm mit einer Gesamtdosis von 50,4 Gy/1,8 Gy auf den Primärtumor oder alternativ einer Dosiseskalation bis zu einer Gesamtdosis von 61,6 Gy/2,2 Gy. Die regionären Lymphknoten wurden in beiden Gruppen mit 50,4 Gy/1,8 Gy bestrahlt. Die simultane Chemotherapie bestand in beiden Armen aus Carboplatin (AUC2) und Paclitaxel (50 mg/m^2^) einmal wöchentlich über 6 Wochen. Der primäre Endpunkt war das lokale progressionsfreie Überleben (LPFS).

## Ergebnisse

Von September 2012 bis Juni 2018 wurden 260 Patient*innen eingeschlossen; 61 % hatten ein Plattenepithelkarzinom, 39 % ein Adenokarzinom. Eine vollständige Strahlenbehandlung (RT) erhielten insgesamt 94 % der Patient*innen, bei 85 % konnten mindestens 5 Chemotherapiezyklen verabreicht werden. Nach einer medianen Nachbeobachtungszeit von 50 Monaten betrug das lokale progressionsfreie Überleben nach 3 Jahren 70 % in der Standarddosisgruppe gegenüber 73 % in der Dosiseskalationsgruppe (Unterschied nicht signifikant). Das LPFS erreichte bei Plattenepithelkarzinomen 75 % bzw. 79 %, und bei Adenokarzinomen 61 % bzw. 61 % nach 3 Jahren (Unterschiede nicht signifikant). Das lokoregionäre progressionsfreie Überleben betrug nach 3 Jahren 52 % im Arm mit der Standarddosis und 59 % in der Dosiseskalationsgruppe (*p* = 0,08). Die Grad-4- und -5-Akuttoxizität erreichte 12 % bzw. 5 % im Standarddosisarm gegenüber 14 % bzw. 10 % im Dosiseskalationsarm (*p* = 0,15).

## Schlussfolgerung der Autoren

Bei Patient*innen mit fortgeschrittenem Ösophaguskarzinom, die mit einer definitiven Radiochemotherapie behandelt wurden, führte eine Dosiseskalation auf 61,6 Gy zu keiner signifikanten Verbesserung der lokalen Kontrolle im Vergleich zu 50,4 Gy. Der fehlende Nutzen der Dosiseskalation wurde für beide Histologien beobachtet.

## Kommentar

Verschiedene Dosierungsschemata wurden für die definitive RCT von Ösophaguskarzinomen untersucht [1]. Historisch zeigte die RTOG-85-01-Studie signifikant verbesserte Gesamtüberlebensraten nach RCT mit 50 Gy/2 Gy im Vergleich zur alleinigen Radiotherapie (RT) mit 64 Gy/2 Gy [2]. Die lokoregionäre Rezidivrate/Progressionsrate betrug hierbei allerdings 47 %. Die hohe Rate an Lokalrezidiven/Progressionen nach definitiver RCT bis 50 Gy ließ vermuten, dass höhere Bestrahlungsdosen die lokale Kontrolle potenziell weiter verbessern würden. Dies führte zur INT-0123-Phase-III-Studie, bei der Patient*innen eine RCT entweder mit 50,4 Gy/1,8 Gy oder mit 64,8 Gy/1,8 Gy erhielten [3]. Auch in dieser Studie zeigte sich kein Unterschied zur lokalen/lokoregionären Tumorkontrolle oder dem Überleben in beiden Armen. Obwohl in der Hochdosisgruppe 11 behandlungsbedingte Todesfälle auftraten, verglichen mit zwei in der Standarddosisgruppe, traten 7 der 11 Todesfälle bei Patient*innen auf, die 50,4 Gy oder weniger erhalten hatten. Relativierend darf aber bemerkt werden, dass die INT-0123-Studie vor der Einführung moderner Strahlentherapietechniken durchgeführt wurde, was die hohen Letalitätsraten zumindest zum Teil erklärt.

In der hier kommentierten, randomisierten Phase-III-Studie ARTDECO wurde nach einer medianen Nachbeobachtungszeit von 50 Monaten der primäre Endpunkt des lokal progressionsfreien Überlebens (LPFS) nach einer Dosiseskalation der Strahlentherapie von 50,4 Gy auf 61,6 Gy auf den Primärtumor nicht signifikant verbessert [4]. Einige Aspekte der Studie sind deshalb hier erwähnenswert. In der ARTDECO-Studie waren die Staging- und Strahlentherapietechniken auf dem neuesten Stand. Das Fehlen eines Vorteils in Bezug auf die lokale Kontrolle nach einer Dosiseskalation über 50,4 Gy hinaus ist daher bemerkenswert. Obwohl in der Dosiseskalationsgruppe ein leichter Anstieg der toxizitätsbedingten Todesfälle beobachtet wurde, erklärt dies nicht die fehlende Verbesserung der lokalen Tumorkontrolle. Die niedriger dosierten Bestrahlungsvolumina deckten die regionalen Lymphknoten im Vergleich zu dem in der CROSS-Studie verwendeten Bestrahlungsprotokoll adäquat ab.

Anders als beim nichtkleinzelligen Lungenkarzinom [5] wird bei Patient*innen mit Ösophaguskarzinom nach wie vor eine Bestrahlung der elektiven Lymphknoten empfohlen, auch wenn im Rahmen des Stagings eine FDG-PET/CT durchgeführt wurde. Es gibt aber zunehmend Hinweise darauf, dass die elektive Bestrahlung von Lymphknoten die Immunantwort gegen den Tumor beeinträchtigen könnte [6–8], obwohl zur Erhärtung dieser Vermutung weitere Studien erforderlich sind. Darüber hinaus wurde die Studie kritisiert, weil ihre statistische Teststärke („Power“) für den primären Endpunkt das notwendige Maß unterschritt („underpowered“), was die Zuverlässigkeit der Ergebnisse beeinträchtigen könnte [9].

## Fazit

Die Verbesserung der lokalen Kontrolle ist nach wie vor ein wichtiges Ziel bei der Behandlung von Ösophaguskarzinomen. Die Evidenz aus den Studienergebnissen von RTOG 85-01, INT 0123 und nun auch ARTDECO spricht beim fortgeschrittenen Ösophaguskarzinom für eine erforderliche Gesamtdosis von 50,4 Gy in 28 Fraktionen als Standarddosis, wenn die Patient*innen mit einer definitiven RCT behandelt werden. Die Ergebnisse der noch laufenden Dosiseskalationsstudien (ClinicalTrials.gov-Identifikator: NCT01348217, NCT02741856 und NCT02556762) stehen noch aus und werden weitere randomisierte Daten zur Frage der RT-Dosiseskalation beim Ösophaguskarzinom liefern.

*Emmanouil Fokas, Daniel Martin, Claus Rödel, Frankfurt/M.*
